# Supplementary material for: Thrombin Induces Angiotensin II-Mediated Senescence in Atrial Endothelial Cells: Impact on Pro-Remodeling Patterns
Source: J Clin Med. 2019 Oct 1;8(10):1570. doi: 10.3390/jcm8101570 (PMC6833093; doi:10.3390/jcm8101570)
Supplement: Supplementary file 1 [file jcm-08-01570-s001.pdf]

Additional Figure 1

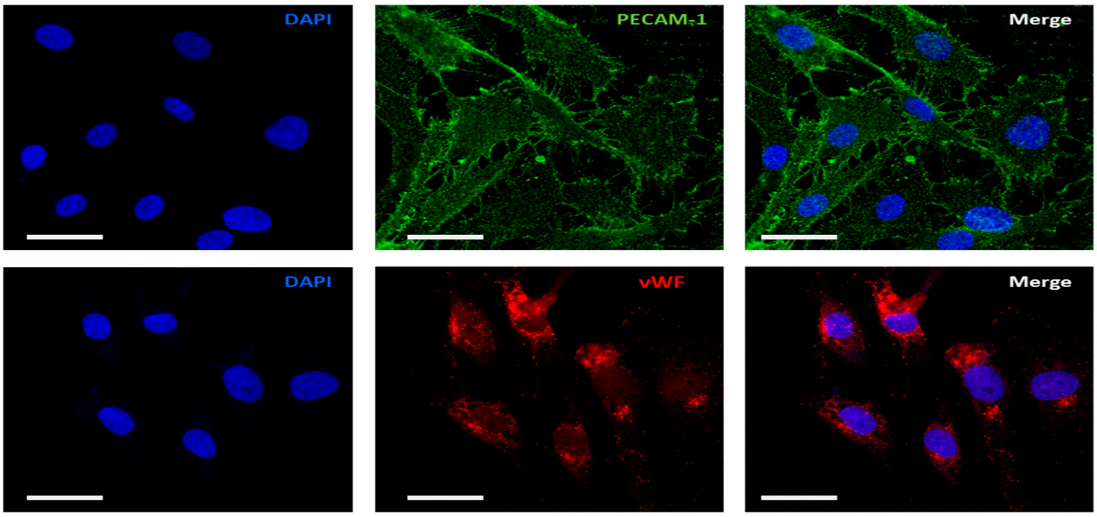

Immunofluorescence staining of endothelial markers in atrial endothelial cells.
Atrial endothelial cells (P1) were cultured in Millicell EZ SLIDE 8-well glass slide (Merck) and fixed with 4% paraformaldehyde. After blocking and permeabilization (PBS + BSA 1% + Triton X-100 0.5 %) cells were incubated with either mouse anti-PECAM-1 (550300, BD Pharmingen; green fluorescence) or rabbit anti-von Willebrand factor (Ab6994, Abcam; red fluorescence) antibodies. Anti mouse IgG coupled to Alexa Fluor 488 conjugate or polyclonal goat anti rabbit IgG coupled to Alexa Fluor 633 conjugate (Invitrogen) were used as secondary antibodies. Nuclei were counterstained with DAPI (blue fluorescence). Images were captured using a Leica SP2 confocal laser-scanning microscope (Scale bar 25 µm).
